# Cutting Force Prediction Models by FEA and RSM When Machining X56 Steel with Single Diamond Grit

**DOI:** 10.3390/mi12030326

**Published:** 2021-03-19

**Authors:** Lan Zhang, Xianbin Sha, Ming Liu, Liquan Wang, Yongyin Pang

**Affiliations:** College of Mechanical and Electrical Engineering, Harbin Engineering University, Nantong Ave 145, Harbin 150001, China; zhanglan@hrbeu.edu.cn (L.Z.); xianbinsha@163.com (X.S.); wangliquan@hrbeu.edu.cn (L.W.); 18804627348@163.com (Y.P.)

**Keywords:** ANOVA, coefficient of friction, cutting force, cutting speed, depth of cut, diamond grit, FEM, RSM

## Abstract

In the field of underwater emergency maintenance, submarine pipeline cutting is generally performed by a diamond wire saw. The process, in essence, involves diamond grits distributed on the surface of the beads cutting X56 pipeline steel bit by bit at high speed. To find the effect of the different parameters (cutting speed, coefficient of friction and depth of cut) on cutting force, the finite element (FEA) method and response surface method (RSM) were adopted to obtain cutting force prediction models. The former was based on 64 simulations; the latter was designed according to DoE (Design of Experiments). Confirmation experiments were executed to validate the regression models. The results indicate that most of the prediction errors were within 10%, which were acceptable in engineering. Based on variance analyses of the RSM models, it could be concluded that the depth of the cut played the most important role in determining the cutting force and coefficient the of friction was less influential. Despite making little direct contribution to the cutting force, the cutting speed is not supposed to be high for reducing the coefficient of friction. The cutting force models are instructive in manufacturing the diamond beads by determining the protrusion height of the diamond grits and the future planning of the cutting parameters.

## 1. Introduction

Submarine pipelines play a major role in the transportation of offshore oil and gas, but they fail from time to time due to various reasons [1]. It is necessary to remove the faulty part for maintenance, yet conventional means are not as effective as desired. In the past few decades, diamond wire saws have become the top choice for cutting hard material [2] and those designed for submarine pipeline cutting are already in practical use [3]. The study of the factors influencing submarine pipeline cutting is of great significance for the planning of the cutting parameters. The reasonable feed speed range of the diamond wire saw cutting submarine pipelines was defined [4]. Considering that both the service life and the working efficiency of the brazed diamond grits are superior to the sintered ones, the former was chosen as subject of this work [5]. The primary wear form of the diamond beads is pulled-out grits, and the breakages of the diamond wire were mainly due to fatigue failure [6,7]. Therefore, the planning of the cutting parameters is crucial to diamond wire saw cutting.

Numerous models were proposed to explain the mechanism of the cutting force [8,9,10,11]. The issues become more complicated in micromachines. Conventional models need adjustment for not being able to capture its behavior in microscale [12,13]. The chip formation mechanism varies considerably [14]. The factors that are significant in micromachines might be of little significance in conventional machining [15]. Chip formation models combining cutting tool geometries and materials microstructures were developed [16,17,18]. The chip formation mechanism of both the single diamond grit and the diamond beads was analyzed in the previous work [19].

FEM has proven to be an effective method of studying friction and cutting force [20,21]. The authors compared various pieces of FEM software and found that the performance of DEFORM-2D is superior to the others in large deformation cutting [22]. FEM was also applied to predict the tool wear evolution and tool life in orthogonal cutting [23]. To derive the magnitude and distribution of stress/strain in the metal matrix material, the authors used ANSYS/LS-DYNA software to simulate the cutting process of SiC particle-reinforced aluminum-based metal materials and the results indicated that the contact between the cutting tool and the particles was the main cause of particle fracture and dislodgement [24]. The experimental work is substituted for virtual experiments carried out using a finite element method model of the cutting process to obtain the specific cutting coefficients [25].

The response surface methodology (RSM) is convenient for predicting the interaction and the main effects of the different influential combinations of the machining parameters [26,27]. The prediction models of the cutting forces were established using the FEM and RSM methods, respectively, and the results indicate that both methods can be used for the accurate prediction of the cutting forces [28]. Besides, it is feasible to use the RSM method to model the cutting force based on the FE data [29]. 

In this work, two means were used to obtain the regression equation of the cutting force. One was to use AdvantEdge software to simulate the single grit cutting steel, the virtual experiment data of which was applied to fit the empirical equation of the cutting force; the other obtained the regression equation of the cutting force and corresponding response surface by Design of Experiments. The effect of the different parameters on the cutting force was analyzed. Finally, experiments were conducted to verify the reliability of the cutting force equations.

## 2. Finite Element Modeling

Virtual experiments using FEM have proved to be an effective means of substituting experiments [25]. The chief advantage of FEM is the convenience of obtaining machining information that is difficult to obtain without massive experiments [30]. The mechanisms of diamond grits cutting and the procedural steps involved in the modeling were discussed as follows: 

### 2.1. The Mechanisms of Diamond Grits Cutting

**Figure 1:**
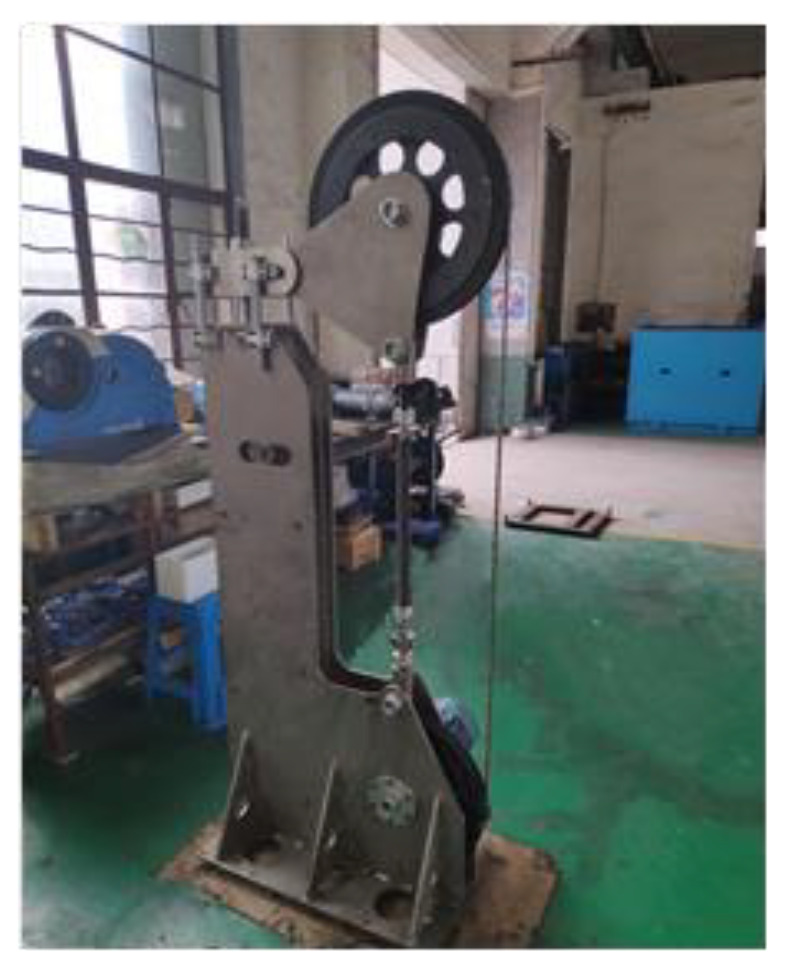
Experimental platform of diamond wire saw cutting.

To analyze the mechanisms of diamond grits cutting, an experimental platform (Figure 1) of diamond wire saw was adopted. 

**Figure 2:**
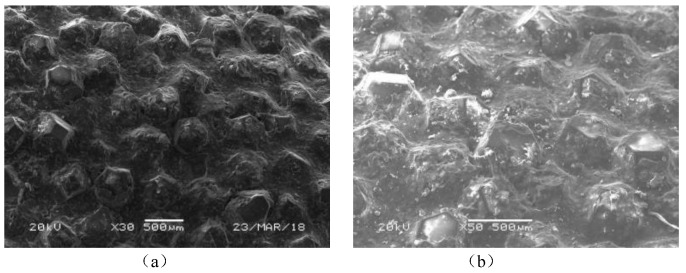
Surface topography of brazed diamond beads: (**a**) before the cutting, (**b**) after the cutting.

In the process of X56 steel cutting, the bond on the surface of diamond grits was worn away and the diamond grits revealed themselves afterward because the matrix of the diamond beads was softer. Figure 2 describes the change in the surface topography of the brazed diamond beads. The initial state of diamond beads’ surface observed by SEM is shown in Figure 2a, and the bond on the surface was worn away after cutting, as shown in Figure 2b.

**Figure 3:**
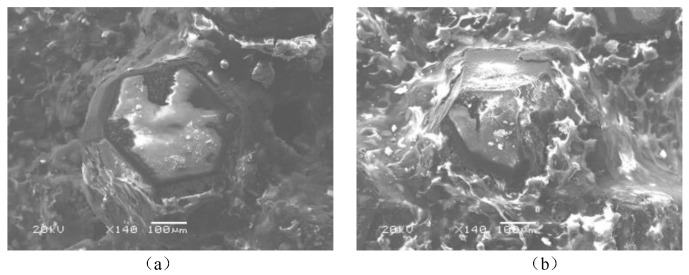
Change of single diamond grit: (**a**) initial state (**b**) abrasive wear and pose change.

Periodic extrusion and friction result in fatigue cracks and abrasive wear on the one hand, yet, on the other hand, subtly changed the positions of the diamond grits, thus avoiding complete abrasion. Figure 3 shows the abrasive wear and position change of a single diamond grit during cutting. The protrusion heights of the brazed diamond beads are higher than those of the sintered ones. Additionally, the bonds between the diamond grits and the matrix of beads are so strong that the dropping of grits is rare. Therefore, a brazed diamond wire saw is an ideal choice for X56 steel pipeline cutting.

**Figure 4:**
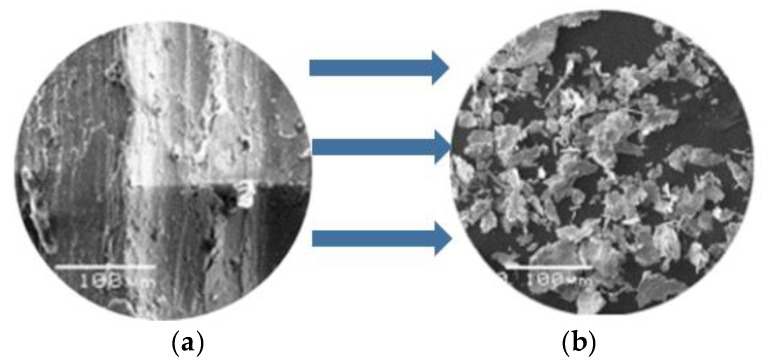
Microscopic observation of the (**a**) grooved surface and (**b**) chips.

The machined chips and the corresponding grooved surface are of significance for the understanding of the cutting process. It would be much too difficult to capture and precisely measure the chips produced by single diamond grits because they are relatively small-scale. Therefore, the chips and scratches produced by diamond beads, instead of single diamond grits, were observed in our previous work [19]. The microscopic observation of the chips and corresponding grooved surfaces are shown in Figure 4a,b, respectively.

### 2.2. Modeling of the Single Diamond Grit

**Figure 5:**
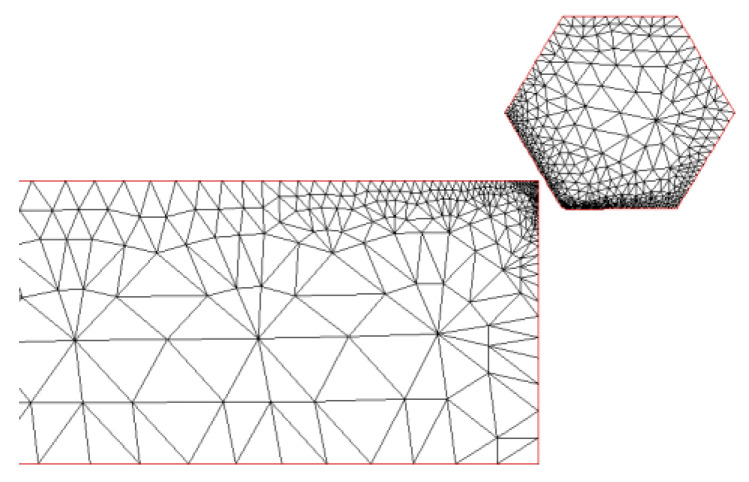
Two-dimensional model of the diamond grit.

The diamond grits on the diamond beads are artificial. According to the diamond beads observed by SEM in Figure 2 and Figure 3, the diamond grits have a relatively regular octahedral hexakis shape, and more than 70% of them are wrapped. The prism length ranges from 150 μm to 250 μm and the value is set as 200 μm for simplicity. The two-dimensional model of the diamond grit is shown in Figure 5.

**Figure 6:**
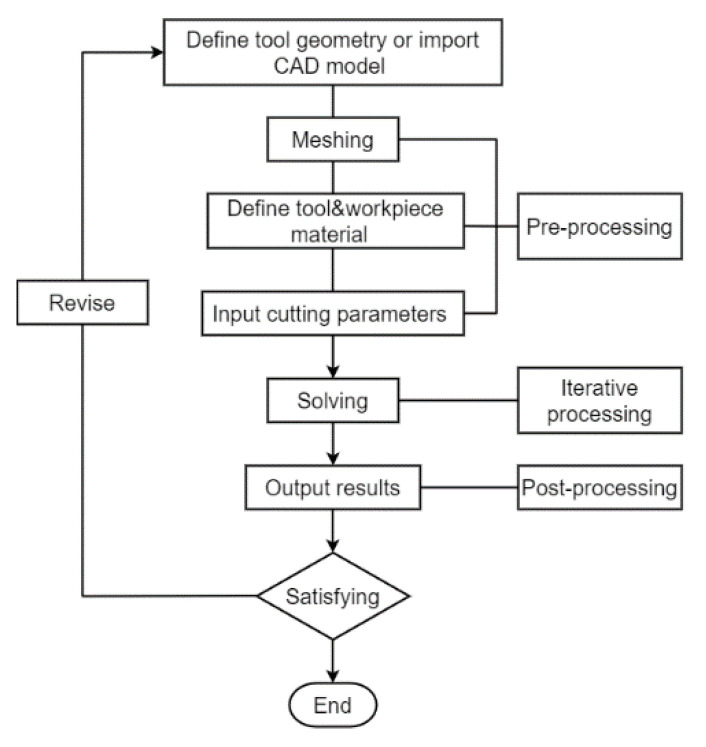
Finite element simulation process diagram of AdvantEdge.

A two-dimensional finite element machining model for X56 steel was performed by AdvantEdge as the 2D machining model has diverged from the 3D machining process [31]. Therefore, the approach features few uncertainties present with this conversion is economical and highly reliable [32]. AdvantEdge is a software designed for metal cutting simulation [32,33], the finite element simulation process diagram of which is shown in Figure 6. Diamond (polycrystalline diamond) was picked as the material of the diamond grits, and X56 steel was chosen as the workpiece material.

### 2.3. Modeling of the Cutting Force

There was an empirical formula describing the relationship between cutting force, friction coefficient, depth of cut and cutting speed. The virtual experiment was conducted by AdvantEdge FEM software to obtain the mathematical model of cutting force.

The range of cutting speed of diamond wire saw is 960–1500 m/min [4], and the cutting depth ranges from 0.01 mm to 0.04 mm. The range of common friction coefficient value is 0.1–0.8. Parameters of the virtual experiments are shown in Table 1, according to which the cutting virtual experiments were carried out to fit the empirical formula and the data is shown in the table of Appendix A.

There is a complex exponential relationship between the cutting force and each factor [34]. During the simulation, it was found that the friction coefficient, cutting speed and depth of cut were the main factors affecting the magnitude of the cutting force. By analogy with the diamond wire cutting model [3], an empirical equation for single grit cutting force can be derived as follows:

(1) $${F\;=\;kv}^{b_{1}}h^{b_{2}}\mu^{b_{3}}$$

where *v*, *h* and μ represent cutting speed, depth of cut and coefficient of friction, respectively. Moreover, ${k,\;b}_{1}$, $b_{2}$ and $b_{3}$ denote constants.

The cutting forces obtained by performing 64 sets of simulations according to the virtual experimental scheme in Table 1 are shown in the Appendix A Table A1 at the end of the paper. According to the mathematical model of the cutting force in Equation (1), the equation obtained by least-squares fitting is as follows:

(2) $$F\;=\;79{.4941v}^{-0.0977}h^{0.7145}\mu^{0.2020}$$

## 3. Response Surface Regression Modeling

### 3.1. Principle of Tribometer and Experiments

**Figure 7:**
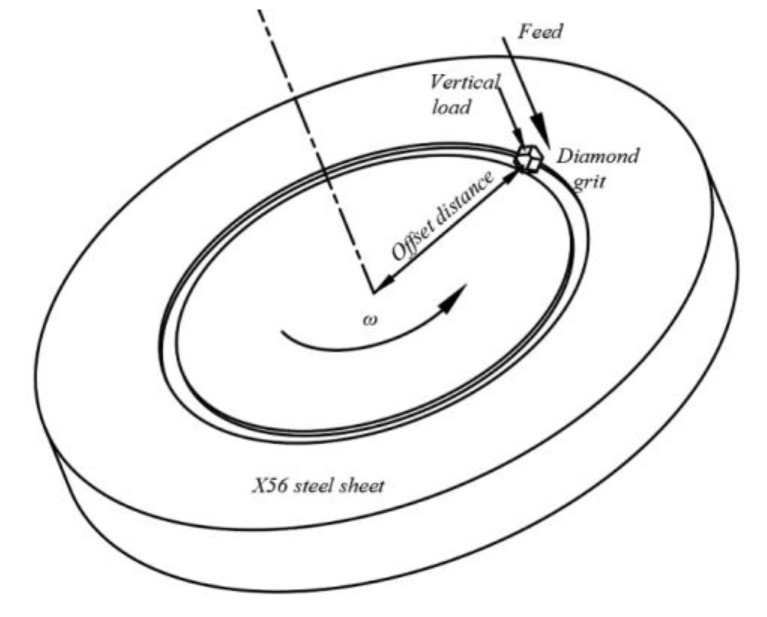
Principle of tribometer.

**Figure 8:**
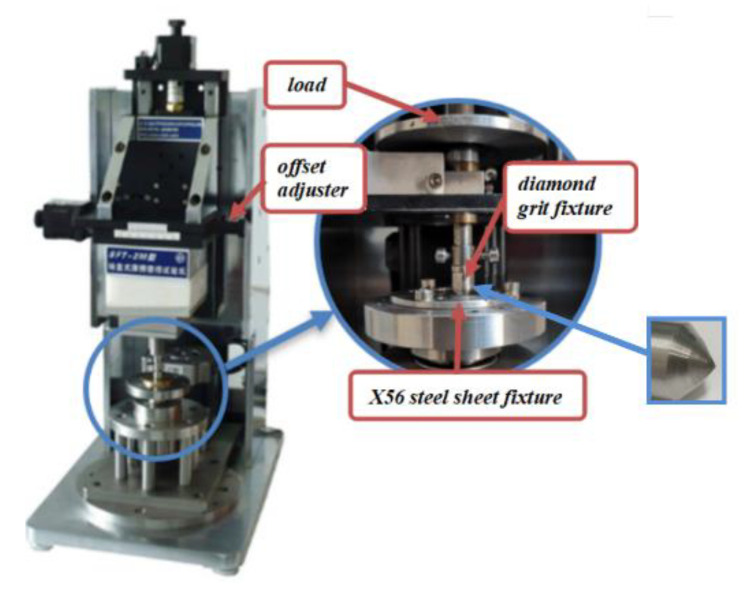
SFT-2M tribometer.

The principle of the tribometer is shown in Figure 7. In the work, the SFT-2M tribometer shown in Figure 8 was used to conduct the experiments providing the data RSM needs.

According to the operation manual, the feed depth of the machine is about 0.01 mm every 10 min under a certain load. The initial feed depth varies as the knob on the top of the machine rotates. The cutting speed is controlled by adjusting the rotate speed ω of the workpiece and the offset distance of the fixture. 

The diamond grits were made of polycrystalline diamond, and the material of the workpiece was X56 steel, The X56 steel was machined into a thin round sheet with a thickness of 2 mm and fixed by the fixture.

**Figure 9:**
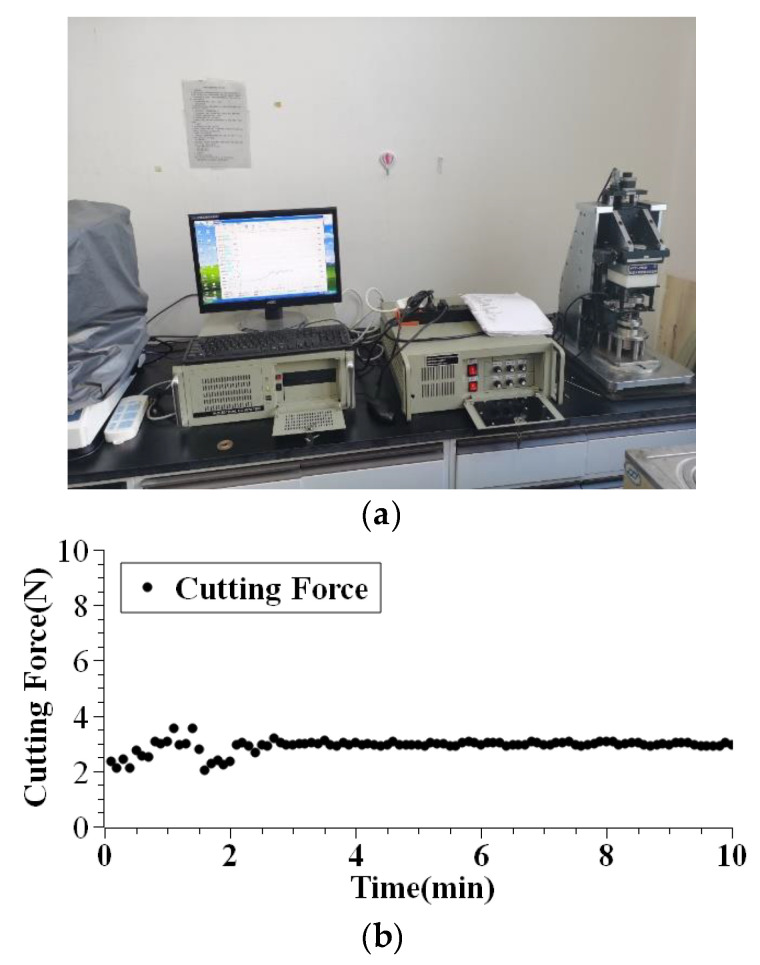
Experimental setup with (**a**) output of SFT-2M tribometer (**b**) cutting force measurement.

The tribometer used for the microcutting experiments was connected to an industrial computer as shown in Figure 9a. The force was output in the form of the curve and sampling points. Figure 9b shows the curve of the output cutting force drawn according to the sampling points. To make it easier for further comparison, the mean value of the small-scale bandwidth fluctuations is chosen as the cutting force.

### 3.2. Response Surface Methodology and Design of Experiment 

The range of friction coefficient and cutting speed is identical to the previous FEM, but the range of cutting depth is modified to 0.02–0.04 mm as oxide film was found on the surface of the steel sheet. Therefore, a certain depth of the steel sheet needs to be removed before the experiment to improve the accuracy.

A central composite face centered design with six center points was used in this study, and the three levels of the experimental input parameters (cutting speed, depth of cut and friction coefficient) are shown in Table 2. The responses obtained after the experiments are given in Table 3. ANOVA analysis was used to obtain significant parameters with their effects, and a response cutting force model was developed. The purpose of this study was to investigate the relationship between the obtained responses and the input variables.

In this work, the mathematical regression equation of the cutting force was found using the response surface method and the relationship between the input parameters was investigated. The experimental results of the cutting force analysis using ANOVA are shown in Table 4.

As can be seen in Table 4, a statistical cubic model is more suitable for analyzing the factors influencing the cutting force of a single diamond grit. The mathematical regression model of the cutting forces generated using Design Expert-12.0 can be expressed as Equation (3).

FrictionForce = 2.79 − 0.0585*A* + 0.646*B* + 0.4895*C* − 0.0131*AB* − 0.0106*AC* + 0.1179*BC* + 0.0079*A*^2^ −0.0306*B*^2^ − 0.1671*C*^2^ − 0.00260*ABC* − 0.0391*A*^2^*B* − 0.0041 *A*^2^*C* + 0.0039*AB*^2^ (3)

where *A* is cutting speed, *B* is depth of cut, *C* is coefficient of friction.

**Figure 10:**
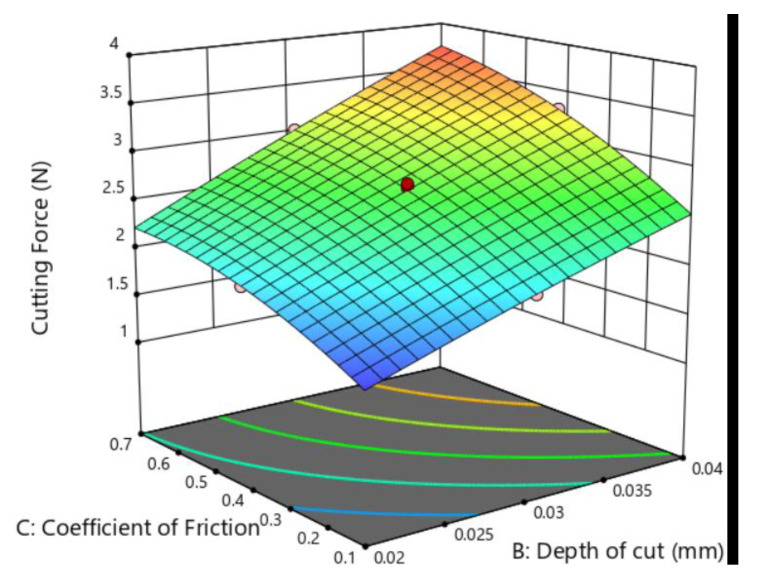
Response surface of the depth of cut and coefficient of friction on cutting force.

**Figure 11:**
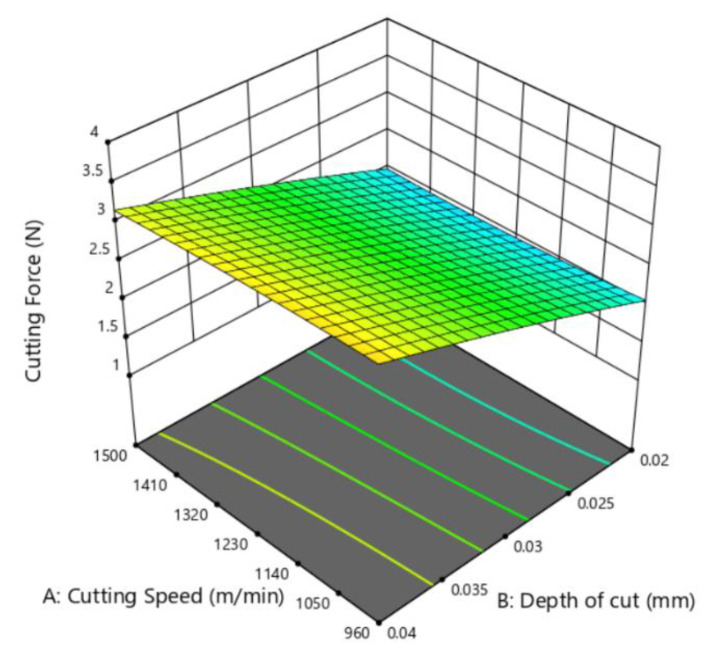
Response surface of the depth of cut and cutting speed on cutting force.

Figure 10 shows the estimated response surface for the cutting force concerning the coefficient of the friction and the depth of the cut. The effect of the depth of the cut and cutting speed on the cutting force is shown in Figure 11. By comparing Figure 10 and Figure 11, it could be concluded that with an increase in the depth of the cut and the coefficient of friction, the cutting force shows an increasing trend. The depth of the cut is the most significant factor that affects cutting force among the three. However, lowering the protrusion height could enlarge the area of the diamond–workpiece contact surface [35]. Therefore, the protrusion heights of the diamond grits deserve high priority when manufacturing diamond beads that serve different purposes. Despite making little direct contribution to the cutting force, the cutting speed is not supposed to be high in the cutting process for reducing the coefficient of friction [36].

## 4. Confirmation Experiment

Besides ANOVA analysis for cutting force by RSM, the tribometer used in the response surface regression modeling was also employed in the validation of the developed models since the instrument was convenient in precise measurement. The confirmation experiment results are shown in Table 5. The comparison between the predicted values for cutting force obtained by RSM, FEA and experimental data indicates that predictions were in close agreement with each other (Table 5). The prediction errors of the FEM model and experimental results vary from −11.7% to −6.4%, while the errors between the RSM model and the experimental results range from −9.99% to 10.02%. The results indicate that the prediction errors of both models are acceptable in engineering [4,37], in view of the randomly distributed diamond grits on the beads in our research [5]. It can also be observed that most of the actual cutting force is less than those calculated by the empirical formula. This phenomenon may be ascribed to the graphitization of the steel, which is not taken into consideration because the diamond wire saw in our research works underwater. 

## 5. Conclusions

Conventional research on the diamond wire saw cutting process concentrated on a macroscale, which was not helpful enough to understand the nature of the machining process, and henceforth, the cutting force prediction model of the single diamond grit was necessary. In this work, the relationship between the cutting force and the different parameters (depth of cut, cutting speed and coefficient of friction) was found through FEA and RSM modelling and experimental substantiation. The subsequent conclusions are as follows:

- Two means were used to obtain the equation of the cutting force. In the first approach, AdvantEdge was used to simulate the cutting process, and the virtual experiment data were applied to fit the empirical equation of the cutting force. In the second one, the regression equation of the cutting force and the corresponding response surface was obtained by Design of Experiments.
- Twelve confirmation experiments were conducted, and the results indicate that both derived models can predict the cutting force with fair accuracy. The prediction errors of the developed models and experimental results vary from −11.7% to 10.02%, which are acceptable in engineering. Additionally, the predicted values of the regression model using FEM were generally lower than the experimental results because graphitization was not included in FEM.
- The results of RSM reveal that with increasing depth of cut and coefficient of friction, cutting force shows an increasing trend. High cutting speed increases cutting efficiency while reducing the coefficient of friction. Hence, the cutting speed needs to be restricted to a specified range. The influence of the depth of the cut is the most significant among the three factors. However, high protrusion contributes to less grit–workpiece contact. Therefore, the protrusion heights of the diamond grits deserve first priority when manufacturing diamond beads that serve different purposes.

In summary, the derived models are effective in the parametric programming of diamond wire saw cutting and manufacturing. 

## Figures and Tables

**Table 1:** Process parameters and their limits.

Levels Parameters	1	2	3	4
Cutting speed (m/min)	960	1140	1320	1500
Depth of cut (mm)	0.01	0.02	0.03	0.04
Coefficient of friction	0.1	0.3	0.5	0.7

**Table 2:** Process parameters and their limits.

Parameters	Level 1	Level 2	Level 3
Cutting Speed (m/min)	960	1230	1500
Depth of Cut (mm)	0.02	0.03	0.04
Coefficient of Friction	0.1	0.4	0.7

**Table 3:** Design layout and experimental results.

Run Order	Cutting Speed (m/min)	Depth of Cut (mm)	Coefficient of Friction	Cutting Force (N)
1	1230	0.03	0.4	2.791
2	1230	0.03	0.1	2.134
3	1500	0.04	0.1	2.55
4	1230	0.02	0.4	2.114
5	960	0.02	0.1	1.66
6	960	0.04	0.7	3.892
7	1230	0.03	0.4	2.791
8	1500	0.02	0.1	1.593
9	960	0.04	0.1	2.659
10	960	0.02	0.7	2.411
11	1230	0.03	0.4	2.791
12	1500	0.02	0.7	2.312
13	1230	0.03	0.7	3.113
14	1230	0.03	0.4	2.79
15	1230	0.03	0.4	2.792
16	1230	0.03	0.4	2.789
17	960	0.03	0.4	2.857
18	1500	0.03	0.4	2.74
19	1500	0.04	0.7	3.73
20	1230	0.04	0.4	3.406

**Table 4:** Results of ANOVA for cutting force by response surface method (RSM).

Source	Sum of Squares	DOF	Mean Square	F-Value	*p*-Value	Significance
**Model**	6.46	13	0.4965	5.566$\;\times 10^{5}$	<0.0001	Significant
A-Cutting Speed	0.0068	1	0.0068	7672.82	<0.0001	-
B-Depth of cut	0.8346	1	0.8346	9.356 $\times 10^{5}$	<0.0001	
C-Coefficient of Friction	0.4792	1	0.4792	5.372$\;\times 10^{5}$	<0.0001	
AB	0.0014	1	0.0014	1544.90	<0.0001	
AC	0.0009	1	0.0009	1012.42	<0.0001	
BC	0.1112	1	0.1112	1.246$\;\times 10^{5}$	<0.0001	
A^2^	0.0002	1	0.0002	192.84	<0.0001	
B^2^	0.0026	1	0.0026	2884.90	<0.0001	
C^2^	0.0768	1	0.0768	86069.91	<0.0001	
ABC	0.0001	1	0.0001	61.80	0.0002	
A^2^B	0.0024	1	0.0024	2745.63	<0.0001	
A^2^C	0.0000	1	0.0000	30.52	0.0015	
AB^2^	0.0000	1	0.0000	26.93	0.0020	
AC^2^	0.0000	0	-	-	-	
B^2^C	0.0000	0				
BC^2^	0.0000	0				
A^3^	0.0000	0				
B^3^	0.0000	0				
C^3^	0.0000	0				
**Residual**	5.352$\;\times 10^{-6}$	6	8.920$\;\times 10^{-7}$			
Lack of Fit	1.894$\;\times 10^{-8}$	1	1.894$\;\times 10^{-8}$	0.0178	0.8992	Not significant
Pure Error	5.333$\;\times 10^{-6}$	5	1.067$\;\times 10^{-6}$	-	-	-
**Cor Total**	6.46	19	-			

**Table 5:** Results of the confirmation experiment.

Numbers of Experiments	Cutting Speed (m/min)	Depth of Cut (mm)	Coefficient of Friction	Cutting Force (N)	RSM Results	Error%	FEM Results	Error%
1	1300	0.02	0.5	1.796	1.996	10.02	2.096	−11.7
2	1000	0.03	0.21	2.211	2.102	5.19	2.411	−8.3
3	1100	0.04	0.51	3.11	2.83	9.89	3.51	−11.4
4	1500	0.02	0.52	1.983	2.203	−9.99	2.083	−6.9
5	1150	0.03	0.48	2.611	2.761	−5.43	2.811	−7.11
6	1240	0.04	0.37	3.001	2.801	7.14	3.251	−7.69
7	1340	0.02	0.61	1.825	1.985	−8.06	1.675	−8.96
8	980	0.03	0.4	2.602	2.5	4.08	2.752	−6.81
9	1280	0.04	0.31	2.927	2.777	5.4	3.127	−6.4
10	1160	0.02	0.49	1.961	1.83	7.16	2.111	−7.11
11	1400	0.03	0.2	2.11	2.311	−8.7	2.31	−8.66
12	1340	0.04	0.35	2.891	3.191	−9.4	3.105	−9.23

## Appendix A

**Table A1:** Results of the virtual experiment.

Num of Experiment	*ν* (m/min)	*f* (mm/r)	*µ*	*F* (N)
1	960	0.01	0.1	0.9178
2			0.3	1.2018
3			0.5	1.4041
4			0.7	1.4081
5		0.02	0.1	1.5144
6			0.3	1.9554
7			0.5	2.3423
8			0.7	2.3861
9		0.03	0.1	2.0514
10			0.3	2.5581
11			0.5	3.0224
12			0.7	3.0845
13		0.04	0.1	2.5614
14			0.3	3.1344
15			0.5	3.5284
16			0.7	3.6851
17	1140	0.01	0.1	0.8922
18			0.3	1.1736
19			0.5	1.3336
20			0.7	1.3224
21		0.02	0.1	1.5044
22			0.3	1.9422
23			0.5	2.2216
24			0.7	2.2826
25		0.03	0.1	2.0472
26			0.3	2.5521
27			0.5	2.9478
28			0.7	3.0292
29		0.04	0.1	2.5468
30			0.3	3.0598
31			0.5	3.4636
32			0.7	3.5468
33	1320	0.01	0.1	0.8794
34			0.3	1.1662
35			0.5	1.3014
36			0.7	1.2626
37		0.02	0.1	1.4896
38			0.3	1.9056
39			0.5	2.2318
40			0.7	2.2416
41		0.03	0.1	2.0496
42			0.3	2.5364
43			0.5	2.9374
44			0.7	2.9072
45		0.04	0.1	2.5484
46			0.3	3.0478
47			0.5	3.4502
48			0.7	3.5606
49	1500	0.01	0.1	0.8728
50			0.3	1.1494
51			0.5	1.2626
52			0.7	1.2232
53		0.02	0.1	1.4896
54			0.3	1.8744
55			0.5	2.1764
56			0.7	2.2094
57		0.03	0.1	2.0292
58			0.3	2.5174
59			0.5	2.8846
60			0.7	2.9058
61		0.04	0.1	2.5443
62			0.3	3.0476
63			0.5	3.4006
64			0.7	3.5346
